# Editorial: Regulation and dysfunction of CSK and CHK

**DOI:** 10.3389/fcell.2023.1254961

**Published:** 2023-07-19

**Authors:** Shudong Zhu, Dianzheng Zhang

**Affiliations:** ^1^ School of Medicine, Nantong University, Nantong, China; ^2^ Department of Bio-Medical Sciences, Philadelphia College of Osteopathic Medicine, Philadelphia, PA, United States

**Keywords:** Csk, Chk, structure, regulation, target, function, apoptosis, integrin

The Src family kinases (SFKs) play indispensable roles in multiple signal transduction pathways. SFKs are activated by autophosphorylation (human Src: Tyr419) in the trans-molecular mode and abnormal SFK activation often leads to cancer and other diseases. C-terminal Src kinase (CSK) phosphorylates SFKs at their C-terminal tyrosine and inactivates SFKs. CSK homologous kinase (CHK) shares same SH2-SH3-SH1 structure and 53% amino acid identity with CSK. There is evidence suggesting CSK may be a dominant player in contrast to an auxiliary role of CHK in certain tissue or cells (Nagy et al., 2020). A unique feature of CHK is that it inactivates SFKs via a non-catalytic mechanism. The recent discovery of CHK promoter hypermethylation-promoted colon cancer supports the notion that dysfunction of CSK/CHK is involved in pathogenesis (Zhu et al., 2021), Although studies have shown that dysregulation of CHK and CSK is present in diseases other than cancers, the underlying mechanisms remain unknown. Moreover, increasing evidence indicates that CHK and CSK are not functionally redundant and likely intertwined with each other in disease development. Recent research suggests that in addition to the CHK/CSK-SFK axis, CHK and CSK likely to be involved in pathways with some novel targets and modes of regulation.

This Research Topic “*Regulation and Dysfunction of CSK and CHK*” consists of 5 review articles contributed by 17 authors in the fields of CSK and CHK. The Research Topic collects different research aspects including molecular structures of CSK and CHK, major signaling pathways such as apoptosis and integrin signaling in cancers affected by CSK and CHK and comprehensive overviews of CSK and CHK highlighting their regulation, molecular targets and functional roles.

In addition to the general features of phosphorylating substrates, CSK and CHK have unique structural motifs determining their substrate specificity and enzymatic efficiencies. CSK and CHK are also subject to regulation by different regulators and/or post-translational modifications. To understand the roles of CSK and CHK and their functional differences in inactivating SFKs, Sun et al. have reviewed the structure-function relationship of CSK and CHK with an emphasis on catalysis, substrate recognition, and the interactions between domain and domain/key phosphorylation site. This review not only summarizes the current understanding of CSK/CHK enzymology but also sheds light on the general mechanism of regulation and catalysis of protein tyrosine kinases. This will be helpful in bolstering protein phosphorylation as a key methodology for the development of anti-cancer therapeutics.

Apoptosis, a form of programmed cell death, plays a key role in multiple biological processes (Capela E Silva and Rodrigues, 2023). The extrinsic pathway is initiated through activation of death receptors (such as Fas, DR4, DR5) engaged by their ligands (such as FasL and Apo2L/TRAIL) which ultimately triggers the activation of caspase 8. The intrinsic pathway is initiated through the activation of the Bcl-2 family of pro-apoptotic proteins such as Bax/Bak in response to pro-apoptotic stimuli. Activation of Bax/Bak leads to increased permeability of the mitochondrial membrane and eventually the activation of effector caspases. These cysteine-aspartic proteases subsequently cleave proteins resulting in cellular demise. Fortner et al. provide an overview of the apoptosis mechanisms induced by CSK. CSK suppresses MAPK, STAT3 and PI3K signaling respectively, all of which are involved in promoting cell survival through upregulation of pro-survival molecules such as Myc and Bcl-2, and downregulation of pro-apoptotic molecules such as Bim and FOXO. The Src-dependent pro-apoptotic effect of CSK has been further confirmed by a novel Src inhibitor (Abdelall et al., 2022). Therefore, targeting key molecules in these pathways to promote apoptosis could be a strategy in the development of potential treatment of diseases such as cancer (Tang et al., 2023).

As a part of the focal adhesion, integrins can sense and send extracellular signals to the cytosol to regulate different processes such as cell migration, invasion, proliferation, and survival. Therefore, integrins play crucial roles in cancer progression and metastasis. Maldonado et al. reviewed how CSK regulates integrin signaling through SFKs. These authors focuse on the crucial role of integrins in sensing and responding to mechanical changes in the surrounding environment by emphasizing their mechanosensing and mechanotransduction functions (Hamidi and Ivaska, 2018; Koudelková et al., 2021). They have also discussed the crosstalk among CSK, integrins, and growth factor receptors suggesting that they could become potential therapeutic targets.

Besides the above specific Research Topic focuses, Zhu et al. have provided overviews of how CSK and CHK are regulated, their molecular targets, and novel biological functions involved. Among many interesting new features of CSK/CHK, it is noteworthy that CHK not only phosphorylates SFKs to inactivate them, CHK also serves as a non-enzymatic inhibitor for many Src family members; Unlike CSK which is expressed ubiquitously, expression of CHK is limited in several tissues; Novel targets of CHK including SHPS-1, paxillin, and synuclein have been identified. Therefore, CHK may complement or strengthen CSK’s effect in inhibiting SFKs in certain tissues. CHK silencing due to DNA methylation (Chüeh et al., 2021; Zhu et al., 2021) in certain cancer types suggests that CHK epigenetics could serve as a therapeutic or diagnostic candidate. On the other hand, in addition to the established CSK-SFK axis, the recent discoveries of novel CSK targets such as MITA (Gao et al., 2020) and novel regulators of CSK such as SPOP (Tawaratsumida et al., 2022), and the unveiled pathology of many diseases newly reported to be associated with CSK warrant further exploration of CSK.

In conclusion, the “Regulation and Dysfunction of CSK and CHK” Research Topic highlights the recent discoveries of molecular structure, targets, regulation and biological function of CSK/CHK. This not only furthered our understanding in basic research but also paved the way for the development of new therapies and diagnosis.
